# Integrated Pest Management for Sustainable Intensification of Agriculture in Asia and Africa

**DOI:** 10.3390/insects6010152

**Published:** 2015-03-05

**Authors:** Jules Pretty, Zareen Pervez Bharucha

**Affiliations:** 1 Department of Biological Sciences and Essex Sustainability Institute, University of Essex, Wivenhoe Park, Colchester CO4 3SQ, UK; 2 Department of Sociology and Essex Sustainability Institute, University of Essex, Wivenhoe Park, Colchester CO4 3SQ, UK; E-Mail: zpbar@essex.ac.uk

**Keywords:** farmer field schools, integrated pest management, social capital, sustainable intensification, resilience

## Abstract

Integrated Pest Management (IPM) is a leading complement and alternative to synthetic pesticides and a form of sustainable intensification with particular importance for tropical smallholders. Global pesticide use has grown over the past 20 years to 3.5 billion kg/year, amounting to a global market worth $45 billion. The external costs of pesticides are $4–$19 (€3–15) per kg of active ingredient applied, suggesting that IPM approaches that result in lower pesticide use will benefit, not only farmers, but also wider environments and human health. Evidence for IPM’s impacts on pesticide use and yields remains patchy. We contribute an evaluation using data from 85 IPM projects from 24 countries of Asia and Africa implemented over the past twenty years. Analysing outcomes on productivity and reliance on pesticides, we find a mean yield increase across projects and crops of 40.9% (SD 72.3), combined with a decline in pesticide use to 30.7% (SD 34.9) compared with baseline. A total of 35 of 115 (30%) crop combinations resulted in a transition to zero pesticide use. We assess successes in four types of IPM projects, and find that at least 50% of pesticide use is not needed in most agroecosystems. Nonetheless, policy support for IPM is relatively rare, counter-interventions from pesticide industry common, and the IPM challenge never done as pests, diseases and weeds evolve and move.

## 1. The Pest Management Challenge and Global Pesticide Use

Pathogens, weeds, and invertebrates cause significant crop losses worldwide, and in doing so present a barrier to the achievement of global food security and poverty reduction. Estimates of the scale of these losses vary by context and scope. Viewed in terms of food security, crops losses to pests may represent the equivalent of food required to feed over 1 billion people [1]. However, the use of synthetic pesticides presents additional challenges and it is now clear that alternative methods need to be applied, which reduce pest damage while avoiding the cost and negative outcomes associated with synthetic pesticides. Integrated Pest Management (IPM) is the deployment of a variety of methods of pest control designed to complement, reduce or replace the application of synthetic pesticides. IPM incorporates the simultaneous management and integration of tactics, the regular monitoring of pests and natural enemies, the use of thresholds for decisions, and spans methods from pesticide product management/substitution to whole agroecosystem redesign. Consequent reduction in the use of synthetic pesticides improves on- and off-farm sustainability, as well as reducing costs to the farmer. IPM systems may also deliver an array of ecosystem goods and services beyond pest control, increasing general resilience at farm and landscape scales. Thus, IPM is an example of sustainable intensification, defined as “producing more output from the same area of land while reducing the negative environmental impacts and at the same time increasing contributions to natural capital and the flow of environmental services” [2,3]. This paper reviews recent evidence of the impacts of IPM in Asia and Africa, and describes its potential to contribute to the sustainable intensification of agriculture amongst smallholders in these contexts.

Pesticides have long been used to control pests and diseases in agriculture [4,5,6,7]. In 2500 BC, Sumerians used sulphur compounds for insect control. Later, seeds were treated by Chinese farmers with natural organic substances to protect against insects, mice and birds, while inorganic mercury and arsenic compounds were used to control body lice. A variety of fumigants, oil sprays and sulphur ointments were used in Greece and Rome, and Pliny recommended the use of arsenic as an insecticide. The widespread use of natural pesticides began in the 17th–18th centuries in Europe: first nicotine and mercuric oxide, then copper sulphate as a fungicide in the early 1800s. By the mid-19th century, rotenone from derris and pyrethrum from chrysanthemum had been discovered, these accompanied by rapid growth in the use of inorganic compounds, particularly of arsenic. Paris Green (copper arsenite) was first used in 1867 and Bordeaux mixture (copper sulphate and lime) was found to be effective against powdery mildew in 1882.

The early part of the 20th century was characterised by the increased use of dangerous compounds of arsenic, cyanide and mercury. Most were broad-acting in their effect on pests and diseases. Some others, such as iron sulphate, were found to have selective herbicidal properties against weeds. Calcium arsenate replaced Paris Green, and by the 1920s arsenic insecticides were widely used on vegetables. The 1930s saw the beginning of the era of synthetic organic compounds, with the introduction of alkyl thiocyanate insecticides, and then the discovery in 1939 of the remarkable insecticidal properties of dichlorodiphenyltrichloroethane (DDT). DDT was followed by the manufacture of other chlorinated hydrocarbons, including aldrin, endrin, heptachlor, and endrin, and the recognition of the herbicidal activity of the phenoxyacetic acids MCPA and 2,4-D. These synthesised products were valued for their persistence in the environment. At the same time, the first organophosphates (OPs), such as parathion and malathion came into commercial use, some found to be toxic to mammals, but all rapidly degrading in the environment to non-toxic secondary compounds. Later generations of compounds included the carbamates and synthetic pyrethroids, both with low toxicity to humans, modern herbicides and fungicides, and lately neonicotinoids that are chemically-similar to nicotine.

While the reporting of pesticide use and market data is patchy and irregular, it is generally clear that the use of synthetic pesticides in agriculture has grown steadily, and now amounts to 3.5 billion kg of active ingredient (a.i.) per year. The highest world market growth rates occurred in the 1960s, at 12% per year, later falling back to 2% and below during the 1980s–1990s, then rising to 3% per year to 2014. The value of the global market is now US $45 billion per year [8,9,10,11,12]. Herbicides account for 42% of sales, insecticides 27%, fungicides 22%, and disinfectants and other agrochemicals 9%. The largest markets are in Europe and Asia (US$12 billion each), Latin America ($10 billion) and North America ($9 billion); the market in the Middle East and Africa is $1.5 bn (all 2012 data). Synthetic pesticides entail huge cost; it has been estimated that the costs to bring a single active ingredient to market are $250 million, having synthesised 140,000 compounds to find each success [13].

China, USA and Argentina now account for 70% of world pesticide use in agriculture (2.44 billion kg of a.i. annually), with China alone now using half of pesticides worldwide (Table 1). Six countries each consume between 50–100 M kg (Thailand, Brazil, Italy, France, Canada and Japan) and thirteen between 10–50 M kg (India, Spain, Germany, Bangladesh, Turkey, South Africa, Russia, Chile, Vietnam, UK, Ghana, Cameroon, and Pakistan). In the past 20 years, there have been substantial increases in pesticide use, with consumption in China growing four-fold, Argentina eight-fold, Brazil three-fold, Bangladesh five-fold, and Thailand four-fold. Countries starting from a low base have seen greater increases: Burkina Faso by 50-fold, Ethiopia 13-fold, Ghana 17-fold, and Cameroon eight-fold. Aggregate use has been stable in Germany and the USA; there have been notable reductions in use in the UK (down 44%), France (down 38%, Japan (down 32%), Italy (down 26%), Vietnam (down 24%) and Denmark (down 21%). Some of these declines were provoked by policy changes, particularly in the EU.

Table 1 also shows some large shifts in the use of insecticides, herbicides and fungicides within the country totals. Several OECD countries have seen large declines in the use of insecticides (Denmark, France, Spain, UK, Japan), herbicides (France, Spain, UK) and fungicides (Sweden, Japan). There has been a large increase in herbicide use in Canada (three-fold), and substantial reductions in insecticide use in India (down 70%). There have been very large increases in both insecticides and herbicides in Argentina, Thailand, Burkina Faso, Ghana, and increases in all categories in Brazil and Chile. There is no published data on category use in China. The example of Thailand shows the rapid growth in use, rising 9% a year from 1.2 kg/ha to 3.7 kg/ha over 1997 to 2007. The largest amounts are used in the intensive vegetable sector (now averaging 13–20 kg active ingredient per ha) [14]. Some 50% of these pesticides are thought to be overused. Pesticides are also used in other sectors, and use can be substantial: the US EPA (2007) reported total use of 510 M kg of a.i. countrywide, of which 63% was in agriculture, 16% in government activities (for weed control on highways and by forest agencies), and 22% in the home and garden [15].

**Table 1:** Country level agricultural pesticide use (1990 to latest data: 2007–2012).

Country	Latest Year (M kg) in Descending Order	Changes in Pesticide Use over an Approximate 20 Year Period (% change)				Data Period
		All Pesticides	Insecticides	Herbicides	Fungicides	
OECD						
USA	386	101%	88%	95%	43%	1990–07
Italy	63	74%	93%	96%	69%	1990–11
France	62	62%	10%	64%	64%	1990–10
Canada	54	172%	103%	171%	335%	1990–08
Japan	52	68%	74%	102%	54%	2000–11
Spain	40	94%	147%	69%	96%	1990–10
Germany	37	113%	71%	102%	95%	1990–11
UK	16	56%	44%	41%	82%	1990–11
Netherlands	8	99%	53%	80%	88%	1990–10
Denmark	4	79%	15%	112%	37%	1990–11
Sweden	1.8	90%	60%	120%	33%	1990–11
Latin America						
Argentina	265	815%	593%	1190%	378%	1993–11
Brazil	76	298%	302%	312%	303%	1991–01
Chile	23	263%	349%	228%	201%	1990–11
Asia						
China	1806	246%	nd	nd	nd	1991–12
Thailand	87	395%	184%	642%	143%	1993–11
India	40	47%	31%	95%	100%	1991–10
Bangladesh	34	489%	2110%	9500%	801%	1990–10
Turkey	33	139%	70%	101%	460%	1990–11
Vietnam	19	76%	57%	97%	151%	1994–01
Pakistan	12	129%	148%	42%	51%	1990–01
Sri Lanka	1.3	91%	137%	54%	112%	1991–11
Africa						
South Africa	27	154%	159%	134%	179%	1994–00
Ghana	15	1683%	591%	5936%	2064%	1995–09
Cameroon	11	766%	582%	1620%	587%	1990–11
Algeria	4	34%	28%	229%	28%	1990–09
Ethiopia	4	1256%	465%	2380%	413%	1995–10
Kenya	1.6	44%	27%	64%	47%	1994–01
Burkina Faso	0.8	4800%	662%	24,800%	nd	1992–11

Sources: [8,9,11]. Note: nd = no data.

In China, pesticide use has increased from 733 M kg in 1990 to 1.806 billion kg in 2012. More than 2.2 billion kg of pesticides are now produced in China, though much is exported. There are 2000 pesticide companies in China [7]. Seven regions each consume more than 100 M kg (Shandong, Hubei, Xinjiang, Hunan, Anhui, Guangdong and Jiangxi), and another ten between 50–100 M kg [9]. The grain output per kg varies more than 10-fold (from 0.1 to 1.3). Use of pesticide a.i. per hectare also varies considerably by country: it is less than 1 kg/ha in India, Canada and across all Africa, 2–3 kg/ha in the USA and France, and 8–10 kg/ha in the Netherlands, China, New Zealand, Chile, and Japan. In the UK, 0.75 kg a.i. were applied per ha in the 1990s; this fell steadily to 0.2 kg in 2012 [16].

The use of synthetic pesticides as the frontline strategy against crop pests has long attracted significant concern, but alternatives, including IPM, have not yet significantly reduced global pesticide use. There is no clear evidence for example that increased adoption of IPM has led to aggregate changes in pesticide use, with the possible exceptions of Vietnam and Denmark. Even here, patchy data on pesticide use makes it difficult to infer precise long-term trends. Policy changes in Indonesia in the 1980s and adoption of IPM and Farmer Field Schools (FFS) might have changed aggregate pesticide use in the early years, but national level data have not been collected since 1993 (between 1989–1993, pesticide use in Indonesia had fallen by 37%). In other contexts, such as in the UK, France, Italy, and Japan, it is likely that the economics of farming has been the main driver of reductions in use, with farmers seeking to cut variable costs where possible. Some changes are also accounted for by the large scale adoption of conservation and no-till agriculture (to approximately 125 M ha in 2012: [17,18] and the adoption of genetically-modified herbicide-tolerant and insect-resistant crops (to some 150 M ha worldwide), the first allowing for greater use of herbicides, the latter reductions in insecticides. At the aggregate level, this has led to large increases in herbicide use in Argentina and Brazil, but no obvious impact in the USA ([19]; though see [20]).

## 2. The Benefits and Costs of Pesticide Use

Pesticides are intended to be hazardous. Their value lies in their ability to kill unwanted organisms. Most act by interfering with biochemical and physiological processes that are common to a wide range of organisms, including, not only pests, weeds, and fungi, but also wildlife and humans. The risks differ from compound to compound, and much of the information on their side-effects remains widely contested. There is no agreed evidence quantifying the harm they do to natural capital or human health, nor a unified view of benefits and costs [21].

Most economic studies assessing the benefits of pesticides are based on a comparison of two scenarios: current use *versus* complete zero. These ask how much reduced pesticide use would cost farmers and the agricultural industry (e.g., [22,23]), and have concluded that substantial costs would arise from yield reductions, the need for additional food imports, and greater consumer risks through exposure to mycotoxins on foods. For the USA, the costs following a complete ban of pesticides were put at $18 billion [22], and 1% of GDP in Germany after a 75% ban [23]*.* Objections subsequently raised to these macro studies centred on the lack of data to describe the relationships between pest/disease ecosystems and the economic system, the reliance on expert opinion, data only derived from research stations, and the failure to account for impacts on human health and the environment (negative externalities) [24,25,26]. Pesticide externalities show features commonly found across the agricultural sector: (i) their costs are often neglected; (ii) they often occur with a time lag; (iii) they often damage groups whose interests are not well-represented; and (iv) the identity of the producer of the externality is not always known [27]. Pesticide externalities thus result in sub-optimal economic and policy outcomes.

Country level externalities have been reported for China (rice cultivation only), Germany, the UK and USA [26,27,28,29,30,31]. Costs are reported for five categories (Table 2), though these are almost certainly underestimates owing to differing risks per product, poor understanding of chronic effects (e.g., in cancer causation or population level effects in ecosystems), weak monitoring systems, and misdiagnoses by doctors of health effects. This framework includes only externalities, those costs passed on to the rest of society through the actions of agriculture. Koleva and Schneider [32] later put the external costs of pesticide use in the USA as much higher (including home and garden use): $12.5 billion annually ($9.5 billion for human health, $3 billion for the environment), and amounting to $42 of costs imposed from every hectare of agricultural land.

Additional private costs borne by farmers themselves are not included, such as from increased pest or weed resistance from the overuse of pesticides, or for training in the use, storage and disposal of pesticides. There also remain unmeasured distributional problems: for example, insect outbreaks arising from pesticide overuse can affect all farmers, even those not using pesticides. There may also be trade-offs: minimum tillage in Asian rice has, for example, resulted in large water savings, reductions in labour needed for land preparation, some yield increases, yet also increases in herbicide use [33].

**Table 2:** Cost category framework for assessing full costs of pesticide use (million US $ per year, 2000).

Damage costs	China ^1^	Germany	UK	USA
1. Drinking water treatment costs	nd	104	215	1059
2. Health costs to humans (farmers, farm workers, rural residents, food consumers)	500–1300	17	2 ^2^	157
3. Pollution incidents in watercourses, fish deaths, monitoring costs and revenue losses in aquaculture and fishing industries	nd	60	7	153
4. Negative effects on on- and off-farm biodiversity (fish, beneficial insects, wildlife, bees, domestic pets)	200–500	10	75	331
5. Negative effects on climate from energy costs of manufacture of pesticides	148	4	3	55
TOTALS	848–1948	195	302	1755

^1^ China costs are just for rice cultivation; ^2^ Does not include any costs of chronic health problems; nd = no data.

The continued use of poorly regulated generics, particularly in developing countries, has led to the “lock-in” of several obsolete pesticides [34]. In Africa, it is estimated that 10% of products used are in World Health Organisation Class 1a (extremely hazardous) and 1b (highly hazardous). These are mostly banned in industrialised countries [35]. Protective clothing is almost unknown, and poisoning incidence and hospitalisation is common in some regions [36]. Jepson *et al.* [35] surveyed 1700 individuals growing 22 crops and using 31 pesticide compounds, and recorded symptoms of cholinesterase inhibition, developmental toxicity, impairment of thyroid function, and depressed red blood cell counts. The externalities of pesticides in Mali are estimated to be 40% of the costs paid by farmers for pesticide products.

Leach and Mumford [26,31] have calculated the costs per kg of active ingredient for the UK, USA and Germany (Table 3). The range of external costs is €3–15 ($4–19) per kg a.i. Earlier research had shown costs for rice cultivation in China to be $3–6.5 per kg (€2.4–5) [26,37]. For Thailand, Praneetvatakul *et al.* [38] calculated the negative externalities of rice to be $19/ha, but rising to $106/ha for intensive vegetables. The active ingredient costs are thus $7.3/kg for rice, and $8.2/kg for vegetables. These costs put crude pesticide externalities worldwide in the range of $10–60 billion (for use of 3.5 billion kg and for a market size of $45 billion). This indicates that the benefits of pesticide use for pest, disease and weed control and their ease of use should be set in the context of the costs of unintended side effects. Such an understanding can thus frame the potential benefits of adopting alternative methods and practices of IPM that produce pest control with less pesticide use.

**Table 3:** Externalities of pesticides for UK, USA and Germany, 2005–06. Source: [31].

Externality category	UK		USA		Germany	
	€ million	€ per kg a.i.	€ million	€ per kg a.i.	€ million	€ per kg a.i.
Pesticides in drinking water	217	9.06	851	2.00	137	5.15
Pollution incidents, fish deaths and monitoring costs	15	0.68	122	0.29	39	1.47
Biodiversity and wildlife losses	23	1.01	157	0.37	5	0.18
Cultural, landscape and tourism	90	3.98	nd	nd	nd	nd
Bee colony losses	2	0.08	109	0.26	2	0.04
Acute effects of pesticides on human health	2	0.08	127	0.30	21	0.80
Totals	349	15.5	1366	3.2	202	7.6

Note: nd = no data.

A recent review of pesticides and impacts on health confirms there are no satisfactory data on pesticide impacts worldwide [39]. A comprehensive analysis of 122 studies published post-2000 showed specific cohorts, especially applicators and farmers, often had significantly higher odds ratios for some cancers, risks of dementias and respiratory symptoms.

Athukorala *et al.* [40] reported that 4%–7% of agricultural workers suffer ill-health from pesticides each year in Sri Lanka, Costa Rica and Nicaragua. When asked, farmers say they are willing to pay to protect against pesticide-induced ill-health, but then generally do not. Pingali and Roger [41] calculated the human health costs of pesticide use in irrigated rice systems of the Philippines, and compared the economics of three pest control strategies: complete protection comprising nine sprays per season, economic threshold decisions involving two sprays per season, and IPM with no pesticides. The complete protection cost most in terms of ill-health and returned the least agricultural returns per hectare (Table 4). Pingali and Roger [41] concluded: “the value of crops lost to pests is invariably lower than the cost of treating pesticide-related illness and the associated loss in farmer productivity. When health costs are factored in, the natural control option is the most profitable pest management strategy”. Finding effective IPM approaches that reduce pesticide use will thus have important effects on rural public health.

**Table 4:** Benefits and health costs of three pest management strategies in irrigated rice, Philippines.

Pest Management Strategy	Agricultural Returns, Excluding Health Costs (Pesos/ha)	Health Costs (Pesos/ha)	Net Benefit (Pesos/ha)
*Complete protection*: standard practice of 9 pesticide sprays per season	11,850	7500	4350
*Economic threshold*: treatment only when threshold passed, usually no more than two applications used	12,800	1190	11,610
*IPM:* pest control emphasises predator preservation and habitat management, alternative hosts and resistant varieties	14,000	0	14,000

Source: [41].

## 3. The Sustainable Intensification of Agriculture

In the past half century, there has been remarkable growth in food production, with increases across the world since the 1960s. During the second half of the 20th century, intensification was the prime driver of increased per capita food production globally. Though total agricultural area expanded by 11% from 4.5 to 5 billion ha, aggregate world food production has grown by 145% (rising to 280% in Asia and 200% in Latin America). The greatest increases have been in China, where a five-fold increase occurred. In industrialized countries, production started from a higher base; yet still doubled in the USA over 40 years and grew by 68% in Western Europe. Over the same period, world population has grown from three to more than seven billion. Again, though, per capita agricultural production has outpaced population growth. For each person today, there is an additional 25% more food compared with 1960 [3].

However, total food production will need to grow again, perhaps by as much as 70% by the time world population has stabilised [42,43,44,45]. The desire for agriculture to produce more food without environmental harm, or even positive contributions to natural and social capital, has been reflected in calls for a wide range of different types of more sustainable agriculture: for a “doubly green revolution” [46], for “alternative agriculture” [47,48], for an “evergreen revolution” [49] for “agroecological intensification” [50], for “green food systems” [51], for “greener revolutions” [52], for “agriculture durable” [53], and for “evergreen agriculture” [54,55]. Others have called for new paradigms to rethink agriculture [56], and for major progress towards sustainable intensification [45]. All centre on the proposition that agricultural and uncultivated systems should no longer be conceived of as separate entities. In light of the need for the sector to contribute directly to the resolution of other global social-ecological challenges, there have also been calls for nutrition-sensitive [57], climate-smart [58] and low-carbon [59] agriculture.

Sustainable production systems should exhibit a number of core attributes [42,60,61]). They should:

- Utilise crop varieties and livestock breeds with a high ratio of productivity to use of externally- and internally-derived inputs;
- Avoid the unnecessary use of external inputs;
- Harness agroecological processes such as nutrient cycling, biological nitrogen fixation, allelopathy, predation and parasitism;
- Minimise use of technologies or practices that have adverse impacts on the environment and human health;
- Make productive use of human capital in the form of knowledge and capacity to adapt and innovate and social capital to resolve common landscape-scale or system-wide problems (such as water, pest or soil management);
- Minimise the impacts of system management on externalities such as greenhouse gas emissions, clean water, carbon sequestration, biodiversity, and dispersal of pests, pathogens and weeds.

Agricultural systems emphasizing these principles tend to display a number of broad features that distinguish them from the process and outcomes of conventional systems. First, these systems tend to be multifunctional within landscapes and economies [54,62,63,64,65]. They jointly produce food and other goods for farmers and markets, while contributing to a range of valued public goods, such as clean water, wildlife, and habitats, carbon sequestration, flood protection, groundwater recharge, landscape amenity value and leisure and tourism opportunities. In their configuration, they capitalise on the synergies and efficiencies that arise from complex ecosystem, social and economic interactions [48].

Second, these systems are diverse, synergistic and tailored to social-ecological context. There are many pathways towards agricultural sustainability, and no single configuration of technologies, inputs and ecological management is more likely to be widely applicable than another. Agricultural sustainability implies the need to fit these factors to the specific circumstances of different agricultural systems [66] Challenges, processes and outcomes will also vary across agricultural sectors: in the UK, for example, [67] found that livestock and dairy operations transitioning towards sustainability had particular difficulties in reducing pollution while attempting to increase yields.

Third, these systems often involve more complex mixes of domesticated plant and animal species and associated management techniques, requiring the deployment of greater skills and knowledge by farmers. To increase production efficiently and sustainably, farmers need to understand under what conditions agricultural inputs (seeds, fertilizers, and pesticides) can either complement or contradict biological processes and ecosystem services that inherently support agriculture [43,68]. In all cases farmers need to see for themselves that added complexity and increased knowledge can result in substantial net benefits to productivity.

Fourth, these systems depend on new configurations of social capital, comprising relations of trust embodied in social organizations, horizontal and vertical partnerships between institutions, and human capital comprising leadership, ingenuity, management skills, and capacity to innovate. Agricultural systems with high levels of social and human assets are able to innovate in the face of uncertainty [69,70,71] and farmer-to-farmer learning has been shown to be particularly important in implementing the context-specific, knowledge-intensive and regenerative practices of sustainable intensification [68,72,73].

Conventional thinking about agricultural sustainability has often assumed that a net reduction in input use, makes such systems essentially extensive (requiring more land to produce the same amount of food). Organic systems often accept lower yields per area of land in order to reduce input use and increase the positive impact on natural capital. However, such organic systems may still be efficient if management, knowledge and information are substituted for purchased external inputs. Recent evidence shows that successful agricultural sustainability initiatives and projects arise from shifts in the factors of agricultural production (e.g., from use of fertilizers to nitrogen-fixing legumes; from pesticides to emphasis on natural enemies; from ploughing to zero-tillage). A better concept than extensive is one that centres on intensification of resources, making better use of existing resources (e.g., land, water, biodiversity) and technologies [3,42,45,48,64,74,75].

Compatibility of the terms “sustainable” and “intensification” was hinted at in the 1980s (e.g., [76,77]), and then first used together in a paper examining the status and potential of African agriculture [78]. Until this point, “intensification” had become synonymous for a type of agriculture characterised as causing harm whilst producing food (e.g., [79,80]). At the same time, “sustainable” was seen as a term to be applied to all that could be good about agriculture. The combination of the terms is an attempt to indicate that desirable ends (more food, better environment) could be achieved by a variety of means. The term was further popularised by a number of key reports: *Reaping the Benefits* [42], *The Future of Food and Farming* [74], and *Save and Grow* [75].

Sustainable intensification (SI) is defined as a process or system where yields are increased without adverse environmental impact and without the cultivation of more land [42]. The concept is thus relatively open, in that it does not articulate or privilege any particular vision of agricultural production [81,82,83]. It emphasises ends rather than means, and does not predetermine technologies, species mix, or particular design components. Sustainable Intensification can be distinguished from former conceptions of “agricultural intensification” as a result of its explicit emphasis on a wider set of environmental and health outcomes than solely productivity enhancement. This further suggests the need for wider sets of indicators to measure impacts [65,84].

IPM delivers on a key principle of sustainable intensification—the use of methods and practices that maintain or improve agricultural productivity whilst both reducing negative impacts and increasing positive outcomes for natural capital and ecosystem services. It, thus, has a role to play in making transitions towards more sustainable systems that can feed a world population that is both growing and changing its consumption patterns [85].

## 4. Integrated Pest Management: Principles and Implementation

IPM emerged after WWII following the recognition that indiscriminate use of insecticide would be ecologically problematic. Early “supervised” and “integrated” control projects were first developed for alfalfa caterpillar and spotted alfalfa aphid [86]. Since then, it has been stated that IPM has become the dominant crop protection paradigm, yet its adoption remains low [87]. As indicated earlier, it has not yet led to a reduction in total pesticide use, nor has it yet eliminated negative externalities. There are also an increasing number of new invasive pests and diseases being discovered, as transfer of species in a globalised world has become easier, and changes in climate and weather patterns have driven shifts in pest and pathogen ranges.

Effective IPM centres on the principle of deploying multiple complementary methods for pest, weed and disease control. IPM has been defined as a “decision-based process for coordinating multiple tactics for control of all classes of pests in an ecologically and economically sound fashion” [86]. This incorporates the simultaneous management and integration of tactics, the regular monitoring of pests and natural enemies, the use of thresholds for decisions, and spans methods from pesticide product management/substitution to whole agroecosystem redesign. This broad range of options allows for many interpretations of IPM [83,87].

IPM approaches can be classified into four main types (Table 5). These vary along a spectrum from targeted or changed use of pesticide compounds to habitat and agroecological design. In only very rare cases, such as the aerial release of the parasitic wasp, *Epidinocarsis lopezi*, to control cassava mealybug in West and Central Africa [88,89], can IPM be implemented without farmer engagement in locally-relevant and effective methods for IPM. In the past 25 years, there has been a substantial increase in understanding on how to increase farmers’ knowledge so that they are able to cultivate and raise crops and livestock whilst reducing or eliminating pesticides. The principles are to find appropriate ways of building the natural, social and human capital in ecosystems such that these provide sufficient services to maintain or increase agricultural productivity whilst reducing or eliminating environmental harm.

The most significant innovation has been the development and deployment of Farmer Field Schools (FFS) from the late 1980s [90]. The aims are education, co-learning and experiential learning so that farmers’ expertise is improved to provide resilience to current and future challenges in agriculture (Table 6). FFS are not just an extension method: they increase knowledge of agroecology, problem-solving skills, group building and political strength [72,91]. FFS have also been recently complemented by modern methods of extension involving video, radio, market stalls, pop-ups, and songs [92]. These can be particularly effective where there are simple messages or heuristics that research has shown will be effective if adopted.

**Table 5:** Typology of IPM.

Type	Examples of Application
1a. Substitution of pesticidal products with other compounds	Synthetic pesticide with high toxicity substituted by another product with low toxicity
	Use of agrobiologicals or biopesticides (e.g., derived from neem)
1b. Management of application of pesticides	Targeted spraying
	Threshold spraying prompted by decision-making derived from observation/data on pest, disease or weed incidence
2. Crop or livestock breeding	Deliberate introduction of resistance or other traits into new varieties or breeds (e.g., recent use of genetic modification for insect resistance and/or herbicide tolerance)
3a. Releases of antagonists, predators or parasites to disrupt or reduce pest populations	Sterile breeding of male pest insects to disrupt mating success at population level
	Identification and deliberate release of parasitoids or predators to control pest populations
3b. Deployment of pheromone compounds to move or trap pests	Sticky and pheromone traps for pest capture
Agroecological habitat design	Seed and seed bed preparation
	Deliberate use of domesticated or wild crops/plants to push-pull pests, predators and parasites
	Use of crop rotations and multiple-cropping to limit pest, disease and weed carryover across seasons or viability within seasons
	Adding host-free periods into rotations
	Adding stakes to fields for bird perches

In Sri Lanka, 610 FFS were conducted between 1995–2002 on farms of mean size of 0.9 ha, and on which paddy rice yields improved slightly from 3.8 to 4.1 t/ha while insecticide applications fell from 3.8 to 1.5 per season [93]. More than a third of farmers eliminated pesticide use completely, and an average farmer could name four natural enemies compared with 1.5 by those who had not attended a FFS. In Burkina Faso, Benin, and Mali, 116,000 farmers have been trained in 3500 FFS for vegetables, rice and cotton [68]. Pesticide use has been cut to 8% (previously, 19 compounds were found in the Senegal Rover, 40% of which exceeded maximum tolerances by 100 fold), biopesticides and neem use has increase by 70%–80%, and there have been substantial increases in yields (e.g., rice in Benin up from 2.3 to 5 t/ha). In Mali, cotton farmers participating in FFS reduced pesticide use by just over 90% compared with pre-FFS use and a control group [94].

FFS and IPM have had an impact at macro level in some countries [95]. Between 1994 and 2007, rice farmers in the Philippines reduced pesticide application frequency and applications per hectare by 70%, increased yields by 12% and increased the inter-year stability of yields. Over this period, national rice production rose from 10.5 to 16.8 Mt [11]. In Bangladesh, pesticide use has increased substantially in recent years (Table 1), and in some districts (e.g., Natore) results in 40–50 applications per season for beans, and 150–200 times on brinjal, sometimes daily [92]. Many farmers spray only crops for market, and keep those for home consumption unsprayed. In other regions of Bangladesh there are severe shortages of rural labour where the burgeoning garment industry has attracted young people, and here FFS have helped farmers adopt and increase herbicide use in rice whilst ensuring that fish and frogs are protected.

**Table 6:** The principal elements of farmer field schools (FFS). Source: [68].

Each FFS consists of a group of 25–30 farmers, working in small sub-groups of about five each. The training is field-based and season-long, usually meeting once per week. The season starts and ends with a “ballot box” pretest and post-test respectively to assess trainees’ progress. Each FFS has one training field, divided into two parts; one IPM-managed (management decisions decided on by the group, not a fixed formula), the other with a conventional treatment regime, either as recommended by the agricultural extension service or through consensus of what farmers feel to be the usual practice for their area. In the mornings, the trainees go into the field in groups of five to make careful observations on growing stage and condition of crop plants, weather, pests and beneficial insects, diseases, soil and water conditions. Interesting specimens are collected, put into plastic bags and brought back for identification and further observation. On returning from the field to the meeting site (usually near the field, under a tree or other shelter), drawings are made of the crop plant which depict plant condition, pests and natural enemies weeds, water, and anything else worth noting. A conclusion about the status of the crop and possible management interventions is drawn by each sub-group and written down under the drawing (agro-ecosystem analysis). Each subgroup presents its results and conclusions for discussion to the entire group. As well as in the preceding field observations, the trainers remain as much as possible in the background, avoiding lecturing, not answering questions directly, but stimulating farmer to think for themselves. Special subjects are introduced in the training, including maintenance of “insect zoos” where observations are made on pests, beneficial insects, and their interactions. Other subjects include leaf removal experiments to assess pest compensatory abilities, life cycles of pests and diseases (and in recent years the expansion of topics away from just IPM). Socio-dynamic exercises serve to strengthen group bonding in the interest of post-FFS farmer to farmer dissemination.

Evaluation of two IPM-FFS programmes in Sichuan, China, found that yields slightly increased whilst pesticide applications fell 40%–50% [96]. In the wet rice agroecosystems, 64% of the insects and spiders were found to be predators and parasites, 19% neutral detritivores, and only 17% rice pests. Beneficials were extremely effective at controlling pests, until pesticides were used.

Nonetheless, it is difficult to overcome the fears that farmers have; often these have been encouraged by the pesticide industry. Farmers need to overcome fears that insects always cause harm, that insect pests will transfer from sprayed to unsprayed fields and farms, and fears of crop loss [97]. In some cases, farmers have experienced anxieties about maintaining good social relations, spraying their crops secretly at night. However, where farmers did join FFS, they were able to have the confidence to make dramatic reductions in pesticide applications from 1.9 to 0.3 per season [72].

FFS have now been used in 90 countries, including in central and east Europe, USA and Denmark [98]. Between 10–20 million farmers have graduated from FFS, including 650,000 in Bangladesh, 250,000 in India, 930,000 in Vietnam, 1.1 million in Indonesia, 500,000 in the Philippines, and 90,000 in Cambodia. By the early 2000s, 20,000 FFS graduates were running FFS for other farmers, having graduated from farmer to expert trainer.

## 5. Analysis of IPM Projects and Programmes in Asia and Africa

There have been relatively few cross-country evaluations of the effectiveness of IPM [60,72,91,93]. These complement wider evaluations of sustainable intensification [2,3,50]; evaluations of organic and resource-conserving agriculture in Africa and Latin America where yields improved in 19 of the 25 cases [99], and comparisons of tropical organic with conventional systems [100].

Here we have analysed 85 IPM projects from 24 countries of Asia and Africa implemented over the past twenty-five years (1990–2014) in order to assess outcomes on productivity and reliance on pesticides. Projects have been implemented in Bangladesh, Cambodia, China, India, Indonesia, Japan, Laos, Nepal, Pakistan, Philippines, Sri Lanka, Thailand and Vietnam; and Burkina Faso, Egypt, Ghana, Kenya, Mali, Malawi, Niger, Senegal, Tanzania, Uganda and Zimbabwe (see Supplementary Table S1).

We have chosen projects as units of analysis as they represent a deliberate attempt to address an existing problem (e.g., a new pest or overuse of a particular compound) or to create a new benefit based on research or knowledge from elsewhere in the field (e.g., new agroecological design). We searched the published literature for evaluations of such projects from Asia and Africa, and identified 85 projects with 115 datasets on combined changes to crop productivity and pesticide use for rice, maize, wheat, sorghum/millet, vegetables, potato/sweet potato, soybean/bean and cotton/tea. The time elapsed from project start to measurement of reported impact varied between 1–5 years. Across these projects, we estimate that 10–20 million farmers on 10–15 M ha have adopted a variety of effective IPM methods and approaches.

In principle, there are four possible trajectories an agroecosystem can take with the implementation of IPM:

- both pesticide use and yields increase (PY);
- pesticide use increases but yields decline (Py);
- both pesticide use and yields fall (py);
- pesticide use declines, but yields increase (pY).

The conventional assumption is that pesticide use and yields are positively correlated, suggesting that only trajectories into PY or py are likely. A shift into Py should be against economic rationale, as farmers’ returns would be lowered, and thus there should be incentives to change practices. A shift into pY would suggest that current pesticide use is inefficient and can be amended.

Pesticide use data is derived from records of changes to the number of sprays per hectare and/or the amount of a.i. applied per hectare. Yield data is recorded as kg/ha per crop. Some data is derived from longitudinal studies (where projects are evaluated before and after) or latitudinal studies (where projects are evaluated with and without intervention). The double delta model involves both latitudinal and longitudinal comparisons, and is considered robust [72]. Evaluations may suffer from placement bias (non-random programme placement through selection of areas or communities easiest to work with or most likely to succeed) and non-random assignment (participation in FFS is voluntary and self-selecting). Ideally, there would be exact matching of farmers with IPM/FFS as a treatment and those without, but again it is usually methodologically impossible to control for a range of factors, such as farm design, off-farm income, age, access to credit, ownership of mobile phones, access to tarmac roads and irrigation, and education level of family members.

It has not been possible to standardise the time elapsed since introduction of interventions to measured impacts (this varies in these projects from one to five years) or the physical distance between with and without treatments (leaving open the opportunity of farmers learning from one another and reducing the difference between with and without treatment). Again inherent is the potential for selection bias: these are all published studies, which will have involved selection of places, people and projects where there have been outcomes worth reporting (whether positive or negative).

**Figure 1:**
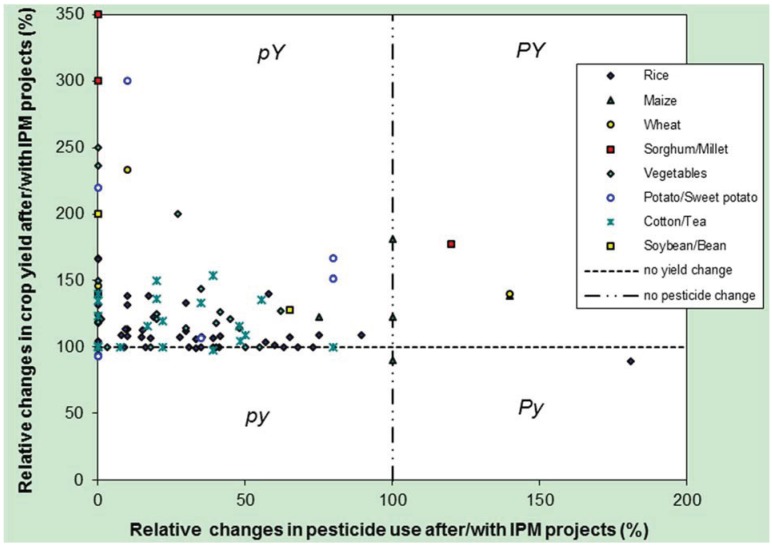
Impacts of IPM projects and programmes on pesticide use and crop yields (data from 115 crop combinations, 85 projects, 24 countries of Africa and Asia).

The mean yield change across projects and crops is an increase of 40.9% (SD 72.3), combined with a decline in pesticide use to 30.7% (SD 34.9) of the original use. A total of 35 of 115 (30%) crop combinations resulted in a transition to zero pesticide use (though none of the projects were formally called organic or involved transitions to organic standards); 18 of 115 (16%) crop combinations resulted in no changes to yields (Figure 1).

We found only one Py case (increased pesticide use combined with reduced yield) in the recent literature, reported by Feder *et al.* [101]. This evaluation considered the impact of FFS training in Indonesian rice cultivation. A number of py (declines in both pesticide use and yield) cases have been reported elsewhere for transitions in high-yield systems typical in Europe following adoption of integrated farming systems. Here, large reductions in pesticide use are accompanied by up to 20% falls in yields [102]. PY contains examples that have involved the adoption of conservation and no-till agriculture, where the reduced tillage improves soil health, reduces erosion, and improves surface water quality, but which require increased use of herbicides for weed control. The rapid growth in herbicide use in Argentina and Brazil (Table 1) has followed the widespread adoption of zero-tillage systems, and these show a transition to PY (though this is not inevitable, as there are organic ZT systems in Latin America: [103]. The majority of cases reported here show transitions to the pY sector (pesticide use falls, yields increase).

While pesticide reductions with IPM may be expected, yield increases induced by IPM are more complex. IPM may, for example, reduce the incidence of severe-loss years, though not increase yields in a normal year, thus increasing mean production across years. Many IPM projects involve interventions focused on more than just pest management (e.g., push-pull systems where legumes fix soil nitrogen as well as help manage parasitoids and depress weeds), or if they involve a significant component of farmer training (e.g., through FFS), then farmers’ capabilities at innovating in a number of areas of their agroecosystems will have increased, such as in soil and water management [94]. Yield improvements may also be accompanied by new income streams that open up with increased flows of ecosystem goods and services. Push-pull IPM systems for example may provide improved livestock feed, enabling smallholders to diversify into dairying and poultry. This in turn provides increased manure for farms (helping soil health and yields), and also translates into increased income and better nutrition. Farmers may also be able to invest cash savings made from reducing pesticide use into better seeds and fertilizers, both of which would increase yields. These arrays of improvements are a good example of the synergistic cycles, which can result from sustainable intensification.

## 6. Lessons Learned from the Four Types of IPM Interventions

Four types of IPM may contribute to sustainable intensification (see Table 5). Here we summarise key lessons learned from these four types.

### 6.1. 1a/1b Substitution and/or Management of Pesticide Compounds

The first type of IPM intervention involves the careful management and targeted use of pesticides, and their substitution with other compounds where possible. An example, now widely used, is biopesticide derived from neem (*Azardirachta indica*) [104].

One highly effective example of changed decision-making arose in irrigated rice systems of SE Asia. There are abundant generalist predators in early season tropical rice fields, and these are lost when sprayed [105]. Nitrogen-enriched fields further lead to increased incidence of herbivorous pest outbreaks. One of the most successful uses of a simple decision-making heuristic was in the Mekong Delta of Vietnam, where participatory research discovered that insecticide applications in the first 40 days of rice planting were ineffective [106]. This was called NES: no early spray for leaf eating insects [107]. Eliminating these sprays saved farmers money, and between 1992 and 1997, two million farmers adopted this practice, reducing pesticide use by over half over a large area. But the advances were short-lived. FFS farmers kept their use of pesticides at approximately 0.5 sprays per season, but non-FFS farmers saw them rise to 2.1 by 2010.

### 6.2. Crop or Livestock Breeding

The breeding of resistance into varieties or breeds has long been a key target for research. There is, though, evidence to suggest that natural resistance mechanisms were often lost or traded-off for yield improvement traits [108,109]. Deliberate breeding of resistance traits can be highly effective, though there are some disadvantages: this approach is not always accessible to small farmers who will have to buy new seeds, the need for researchers to predict and identify target pests or disease in advance, and the need to continue to produce new varieties, as large areas planted to resistant varieties will always put heavy selection pressure on resistant pest and disease traits to emerge in natural populations. In Uganda, the new disease threats of cassava mosaic virus and brown streak virus had led to collapse in production. Three innovations were effective: disease-free planting materials, improved cultivation methods and post-harvest processing (to increase income for women’s groups). These substantially increased yields, not least because new materials were early maturing in 6–12 months [110].

The past twenty years have seen the emergence of IR and HT varieties of maize, cotton, soybean and canola produced by genetic modification. Some of these have been widely adopted in India, China and Burkina Faso. *Bt* crops produced by genetic modification are simply a form of host-plant resistance (HPR) [19]. *Bt* crops have been successfully incorporated into some IPM regimes, resulting in the USA in large reductions in insecticide use on cotton from 5.5 to 2.3 applications per hectare. By 2012, 95% of cotton acreage in the US was *Bt*, herbicide-tolerant or stacked (both traits). In maize across 19 states, the amount of active ingredient applied has fallen from 4100 tonnes in 2001 to 726 t in 2010. The world area of four crops planted to GM traits is now 50% of cotton, 23% of maize, 27%of canola and 66% of soybean [19]. However, the number of weed species with resistance to glyphosate has now risen to 19 (11 in the USA) [20].

### 6.3. 3a/3b Release of Predators or Parasites and/or Deployment of Pheromones

This category of IPM involves classical biological control, where a usually-invasive pest is controlled through the discovery, import and release of a predator or parasitoid. The number of successful examples in agriculture is been limited, but includes the introduction of the parasitoid wasp (*E. lopezi*) from Latin America to control cassava mealybug in West and Central Africa [88], and more recently natural enemies to control papaya mealybug in Asia and millet head miner in West Africa. In China, the pests of the economically-important 4 M ha of brassicas (diamondback moth, cabbage white butterfly, aphids) are controlled by both the release of biocontrol agents and reductions of pesticide use by 20%–70%; and on 1.7 M ha of citrus, companion plants in orchards are grown along with the release of predatory mites (*Amblyseius* spp) [104].

Pearl millet head miner emerged as a major pest in Mali, Burkina Faso, and Niger in the 1970s. With widespread pesticide control not an economic option, and plant breeding unsuccessful over the years, biological control was explored as a possibility, with a natural enemy, the parasitic wasp (*Habrobracon hebetor*), eventually discovered in Senegal [111]. Following a long period of testing, wasp-rearing and release was begun in 2006. Parasitoid kit bags were given to farmers, each containing millet grain, 25 pest larvae and two pairs of *H. hebetor*. Farmer field schools have been run for 700 farmers to increase their engagement and understanding of the approach, and in 2009 a total of 395 villages had become part of the programme, with 700,000 farmers benefiting from the presence of the parasitoid. Yields have been improved by 40%, with kill rates of 72% recorded for the pest larvae. The next phase of the project is targeting more releases, conducting more FFS, and aiming to cover one million hectares of farmland with sustained presence of the parasitoid.

Now increasingly common, pheromone compounds to trap pests are coming into use. In Bangladesh, melon fly (*Bactroccra cucurbitae*) damage of melon, cucurbits and gourds was addressed through 47,000 Farmer Field Schools between 2003–2014 [112,113,114]. In this case, there has been rapid adoption of a simple technology: the use of new pheromone traps using cuelure (a male lure). This is put in a recycled plastic jar or bottle with a small amount of dichlorvos insecticide. Yields have risen 40%–130%, insecticide use fallen from 15 sprays per season to zero, and returns risen 300%.

### 6.4. Agroecological Habitat Design

Type 4 IPM seeks to make positive use of the ecosystem services that can be delivered by agricultural landscapes and habitats. This category of IPM involves the deliberate manipulation of agroecosystems by increasing diversity of in space and/or time. Domesticated or wild crops/plants are used to push or pull pests, predators and parasitoids, or to break rotation patterns to introduce host-free periods. Other approaches include seed and plant preparation, addition of perches to fields to encourage insectivorous birds to settle and feed; use of yellow citrus ants to prevent citrus fruit fly damage. The first such biological control was recorded in in China in 304 AD [104], and many examples since then of deliberate use of ants, frogs and birds for insect pest control. In this sense, agroecology is not an input substitution approach, but about redesigning systems and increasing agroecological integration [73].

Garbach *et al.* [115] use the term “nature gaps” to identify how agricultural systems should promote more positive outcomes for natural capital (analogous to yield gaps where the full potential of a system has not been realised). Agricultural systems should positively contribute to biodiversity habitat, carbon sequestration, climate resilience, energy production, pest control, water quality and quantity, and dietary diversity. This category of IPM project delivers a diverse and comprehensive portfolio of social-ecological “goods” by filling “nature gaps”. Conventional pest-control by contrast, is both a response to “nature gaps” and widens these over time.

A notable example of redesign is the development of the push-pull (*vutu sukumu*) system in East Africa [116,117]. Push-pull involves the behavioural manipulation of insect pests and natural enemies in order to make some areas unattractive and to pull beneficials into crops. The components are usually non-toxic [118]. Push-pull was first conceived in Australia by Pyke *et al.* [119] and the methods comprise visual attractions, use of non-host volatiles (e.g., of citronella, eucalyptus, anti-aggregation pheromones, alarm and sex pheromones, and anti-feedants (e.g., from neem). The success of push-pull principles in IPM has partly emerged from a research understanding of the roles of herbivore-induced plant volatiles (HIPVs), the semiochemicals produced by plants when under attack (e.g., farmasene released by cereals to deter aphids) [118].

In East Africa, legumes and grasses are used to attract and repel parasites and pests of maize (Box 1). These systems have been adopted on 30,000 farms, and followed the initial scientific discovery of the role of semiochemicals released by plants and how they modified insect behaviour [116,120]. It has been shown in the USA that genetic improvement of maize varieties has eliminated some naturally-occurring semiochemical traits. In earlier varieties, roots of maize attacked by corn rootworm emit volatile organic compounds (e.g β-caryophyllene) that attract soil-dwelling nematodes to infect the pest. Restoration of this genetic capacity results in reduced root damage [108,109]. This has been termed influencing the “signal landscape” of crop production systems.

The East African model involves vegetation diversification, intercropping to disrupt host location, molasses grass and desmodium legume that release repellent HIPVs, molasses grass that attracts parasitoids, and trap crops of napier and sudan grass. This results in the stem borer pest of maize being repelled by non-host intercrops, then concentrated into trap crops where there is heavy oviposition and the grasses themselves producing gummy substances that prevent larval development. The desmodium has also been found to suppress Striga (witchweed) development. Push-pull itself has positive side effects, with soils improved, livestock productivity up from the increased availability of fodder. Some farmers have now been using push-pull for 15 years with many innovations emerging (e.g., using upland rice intercropped with desmodium) [117].

Some programmes do not set out to address a pest problem, but achieve pest suppression as a result of changed agroecological habitats. For example, the Fertilizer Tree Systems (FTS) of Malawi, Tanzania, Mozambique, Zambia, and Zimbabwe convert continuous maize cultivation to mixed systems with a variety of nitrogen-fixing shrubs. The FTS address soil fertility problems and reduce dependence on costly fertilizers, producing systems that double maize yields and suppress Striga as a side effect [121]. Some 300,000 smallholders have adopted FTS in southern Africa. As a result of recent regreening efforts across the Sahel, some three million hectares have been improved with the planting of 120 million trees [122,123]. Agricultural and landscape diversity have improved, producing a more resilient landscape [123] that is likely to have a positive impact on populations of predators and other beneficials. In each of Niger and Burkina Faso, some 250–300,000 hectares of degraded land have been improved, causing water table rises and substantially reduced firewood collection workload for women.

**Box 1. Push-pull integrated pest management in East Africa. Source: [116].**

Semiochemicals are compounds released by plants that modify the behaviour of insects (by acting like pheromones). The stemborer is a major pest of maize, and its rising incidence has coincided with the increasing cultivation of maize as a monoculture. Researchers at ICIPE and Rothamsted found that (i) fodder and soil conservation grasses (e.g., Napier grass (*Pennisetum purpureum*) and Molasses grass (*Melinus minutifolia*)) attract stemborers to lay eggs on the grass rather than maize, (ii) legumes such as Desmodium act as repellents, driving the stemborers away. Desmodium also fixes up to 100 kg N ha^−1^ yr^−1^, and releases root allelochemicals that induce abortive germination of the parasitic weed, Striga. Napier grass also releases semiochemicals at a 100 fold greater rate in the first hour of nightfall, just as the stemborer moths seek host plants for oviposition: when the eggs hatch, 80% die as the napier grass also produces a sticky sap that traps the larvae.

Mixed systems were redesigned for farmers, with maize-legume intercrops surrounded by trap crops of grasses. The social infrastructure was redesigned to encourage engagement with farmers through field days, Farmer Field Schools, farmer teachers, mass media, and public meetings. Yields of maize have increased from 1 to 3.5 t ha^−1^, and sorghum from 1 to 3 t ha^−1^. The number of farmers using push-pull has increased from a few hundred to 30,000 in a decade. The target for 2015 is 50,000 hectares covered.

### 6.5. Combinations of Types 1–4 in Integrated Programmes

It is rare that one single intervention results in successful control of a pest, disease or weed. More commonly, a package of technologies and practices must be developed in partnership with local farmers so that these are fitted to local circumstances whilst at the same time increasing farmers’ knowledge through participatory research [112,113,124,125]. Table 7 shows two examples of IPM packages developed through Farmer Participatory Research (FPR) and FFS for onion-shallots in India and green tomatoes in East Africa.

**Table 7:** Components of two IPM packages for onion-shallots in India and green tomatoes in East Africa [114,125].

Onion-shallot IPM package, Tamil Nadu	Tomato IPM package, East Africa
Healthy seed-bulb selection Seed treatment with neem biopesticide Soil applications of Pseudomonas and *Trichoderma* biopesticides Cultivation of barrier crops of maize Sticky traps and pheromone sprays Spray applications of biopesticides Last resort: synthetic pesticides	Soil preparation with *Trichoderma*, neem cake and VAM Seed selection Seed treatment Seedling nursery and grafting Rogueing weeds within 45 days Neem or mustard oil cake in soil Mulching of soil Sticky traps and pheromone sprays Host free period Staking of plants Biological control with parasitoids *Trichogramma* and *Brancon* spp.

Both programmes use a combination of seed and plant preparation, habitat management and amendment, treatment with biopesticides, releases of parasitoids and deployment of sticky and pheromone traps for pest capture. These are implemented with field days, exhibitions, radio programmes, demonstration and field research and experiments. In Tamil Nadu, these reduced the incidence of pests and diseases (onion thrip, leaf miner, *Fusarium* basal rot and purple blotch) by 36%–57%, raising bulb yields from 10.7 to 13.8 t/ha. Onion is a very important commercial crop on some 750,000 ha in India, yet the use of 6–9 pesticide sprays per season had induced resistance in thrips and eliminated natural enemies of leaf miners.

Further examples include tomato IPM in Mali/Senegal where a new viral pest emerged in the early 2000s, transmitted by sweet potato fly (*Bemisia*). The IPM methods include host-free periods to avoid tomato production when whitefly incidence is high and identification of traditional varieties with resistance, and reduced use of synthetic pesticides [124]. Sprays have fallen from 7–10 to 2–3 per season, farmers have saved $200 per ha (costs fell from $285 to $85) and yields have risen from 3.3 to 17.5 t/ha. Farmer individual gains amount to $3300 per ha.

In Nepal, women’s groups use drip irrigation, biofertilizers, biopesticides, bagging, mulching and pheromone/soap-based insect traps on cabbage and cucumber, resulting in longer growing periods and no longer any need for synthetic pesticides [114]. In Uganda, green tomatoes are a high value crop but suffer onion thrip and late blight damage, resulting in 15–25 sprays per season. IPM using sticky yellow traps, resistant varieties, stakes and trellises to keep fruit off the ground and reduce moisture have doubled yields and reduced pesticide use by 75% [126]. Rice and vegetable IPM in Bangladesh includes ten components: resistant varieties, sweeping pests of crops with hand nets, branches for birds in fields, hand weeding vegetables, sawdust burning in vegetable beds, use of poultry refuse, mashed sweet gourd traps in fields, reduced pesticide spraying, grafting of eggplant, and releases of beneficials [127].

## 7. Policy Interventions to Support the Spread of IPM

A key question regarding the sustainability of IPM interventions centres on cost and the long-term impact on farmers knowledge and capability to innovate. A fourteen country review by Braun and Duveskog [98] of FFS showed that costs of a FFS run through a whole season amounted to a mean of $8.70 per participant. Bartlett [127] indicated that a whole FFS cost between $150–1000 for 30 participants. These not only increased yields, but resulted in mean savings of $40 per hectare from reduced pesticide use. It is difficult to imagine public policy interventions with such a low individual cost yet producing lasting changes to social capital, farmers’ knowledge and agricultural outcomes. More recently, FFS in Bangladesh have been shown to create $4 of benefits for every $1 spent [128,129]. This includes the benefits of reducing negative externalities of pesticides (which most cost-benefits studies do not include). Others are more sceptical. For example, Feder *et al.* [101] were sceptical about high up-front costs of IPM-FFS, and considered FFS to be financially unsustainable. However, their consideration of costs did not consider the saved externalities ($3–15 per kg of active ingredient not used), and they did not take account of the shift from hazardous to less-hazardous products [91].

The FAO-IPPM programme has trained 160,000 farmers in Benin, Burkina Faso, Guinea, Mali, Mauritania, Niger and Senegal [68,94]. This centred on two concepts: the importance of beneficial insects and the ability of plants to compensate for pest damage without pesticide interventions. Many farmers have substituted neem products (from the tree *A. indica*) for synthetic pesticides. In these programmes, FFS costs have amounted to $24 per farmer, but have created net benefits of $264 per participating farmer. In the one region of Bla in Mali, farmers saved $470,000 on synthetic pesticides, spent $87,000 on neem products, netting some $380,000 of benefits.

The first FFS was run in Indonesia in 1989; over the next 16 years two million farmers were trained. FAO alone spent $100 million on grants for IPM in Asia over this period [128]. The first FFS in Africa were held in Kenya, Tanzania, and Uganda in 1999 [130]. Within a year, 1500 FFS had been run in Kenya alone. To date, FFS have been held in 90 countries [94], and in all cases there have been increases in income (through reduced costs) and/or improved crop productivity.

Many believed IPM to be too complex for farmers to understand—indeed this is the very reason suggested as the explanation for IPM not spreading widely in industrialised countries [87]. Van den Berg and Jiggins’ [72] review of 25 FFS projects showed that as farmers’ knowledge increase so too did the capacity to reduce pesticide use, with steady falls in rice in Indonesia, Bangladesh, Vietnam and Sri Lanka. Knowledge grows over time after FFS [131]. In Sri Lanka, FFS farmers were found to continue to innovate more after the programme, taking ideas and investments into house building, sewing machines, fridges, grinding machines, and shop and vegetable outlets [91]. Elsewhere, there is evidence that farmers continued to innovate after their training season, such as investing in early ploughing or hand collecting egg masses to control stem borers [92].

However, despite evidence for the cost-effectiveness of IPM programmes and growing farmer capability and knowledge, explicit policy support for IPM has been relatively rare. The most significant was the Indonesian government actions of the mid-1980s, when 57 insecticides that caused brown planthopper outbreaks were banned, subsidies costing $100 million per year removed, and a shift to IPM with a three-year FAO programme launched. By the end of the 1980s, pesticide imports had fallen by two-thirds, and FFS established that would go on to train 1.5 million farmers in Indonesia alone. Following the success of IPM FFS programmes in Vietnam, where pesticide use fell dramatically and yields increased, the government signed a directive in June 2009 on “Strengthening Pesticide Management.” This policy set out proper disposal of products, tank infrastructure, education of pesticide sellers, and community involvement in reducing pesticide risks and the use of IPM to create healthy crops [95].

The most common interventions have been not necessarily to encourage IPM, but just to limit the use of certain compounds. China recorded 108,000 cases of pesticide poisoning over a six-year period from 1997–2003, of which 7500 resulted in deaths [7]. This led to the banning of key organophosphate compounds in 2007. Previously the Chinese Ministry of Agriculture had implemented in 1975 a “Prevention first then integrated control” policy. Over 1300 species of natural enemies were identified, and efforts at ecological engineering incorporated into rice IPM. There has been widespread mass rearing of *Trichogramma* wasps (especially for the control of maize pests) on 266 M ha in 2012. The consequence of year on year releases provides good IPM control, especially in Heilingjiang, Jilin and Liaoning provinces.

In the EU, Directive 91/414/EE1 2010 resulted in the withdrawal of 60 active ingredients from the European market. IPM was made compulsory from 2014, yet with a quarter of the world market in pesticides in Europe, it will be difficult to make an effective transition from the sole use of pesticides for pest/disease control. Chemical ecology and the knowledge of semiochemicals offer opportunities as does engineering large-scale heterogeneity into a pest-suppressive landscape in the form of buffer strips, hedgerows and wildflower borders [1]. Some European countries have included taxes on pesticide prices: in Sweden at €3/kg, Norway at €2–20/kg and Belgium €0.39/kg [132]. These are generally established to raise resources for cleaning up or treating symptoms, not necessarily being set at levels that would causes farmers to decrease use.

There is little evidence that IPM has been well-established in North American and European agriculture [86]. For many farmers it is still considered time-consuming, complicated, requires the need to hire external consultants, whilst pesticides themselves still represent a cheap insurance policy. There is no reason why the pesticide industry should seek to change such perceptions. In some cases, the relative cost of cheap synthetics may hinder the adoption of alternatives even when farmers are convinced of their effectiveness. In one notable example, biological control methods for IPM in soybean in Brazil were shown to be effective in the field, but were abandoned by farmers as insecticides were easy and cheap. The failure of the IPM project led to an increase in applications of pesticides and more pest resurgences [133]. In an IPM programme in Uttarakhand, India, increasing pesticide use has resulted in higher costs and the loss of no market advantages [134]. Other problems which may explain relatively anaemic adoption rates are the need for collective action at landscape scale and “pesticide industry interference” [87]. Bottrell and Schoenly [135] indicate that the “chemical industry conducts major mass media campaigns to promote insecticidal control in rice throughout Vietnam’s Mekong Delta.” There is also evidence that pesticide companies have appropriated the FFS model to promote greater use of pesticides. There are good reasons for such push-back. IPM-FFS have shown local impacts on pesticide markets. For example in East Java, one impact of FFS was a fall in the number of pesticide sellers from 45 to 3 over six years in the 1990s [91].

For policy makers however, there are compelling reasons for preferring IPM-led approaches. In Mali, there are four million cotton farmers who provide 50%–75% of national export earnings. Over the eight year period of FFS implementation, there has been an increase in farmers trained and diffusion to non-trained, simple technologies adopted for IPM, costs lowered to 2%–5% of previous levels, lower toxicity of products used and found in surface water, and increases in social capital [94]. The challenge remains, however, to find effective ways of transmitting information about IPM to very large numbers of farmers [129]. In Bangladesh, half of the population of 150 million is agricultural with 0.5 ha per family or less. More than 4600 FFS have been conducted at a cost of $20 per FFS, reaching some 165,000 farmers. Other dissemination methods have been use: media on paper and on the internet, field days, extension agents, with growing opportunities to use mobile phones further, yet the challenge remains huge.

Pimentel and Burgess [136] have contended that it is possible to reduce pesticide use in the USA by 50% or more with no impact on yields. Our review of 85 IPM projects and implementation also shows that at least half of the pesticide used in Asia and Africa does not need to be used. The financial case for public intervention in supporting IPM is strong.

## 8. The Continuing Ecological Challenge for IPM

The final reason for active intervention is that the IPM job is never done. Ecological and economic conditions change. Pests, diseases, and weeds evolve, new pests and diseases emerge (sometimes because of pesticide overuse), and pests and diseases are easily transported or are carried to new locations (often where natural enemies do not exist) [137]. New pests that have emerged in the past two-three years include banana leaf roller (Nepal), invasive cassava mealybug (SE Asia), cucumber mosaic virus (Bangladesh), tomato yellow leaf curl virus (West Africa) and cassava mosaic virus and brown streak virus (Uganda).

The papaya mealybug (PM) is a native of Mexico. It spread to the Caribbean in 1994, to the Pacific Islands by 2002, and was reported in Indonesia, India and Sri Lanka in 2008 [138]. In these new locations, there is an absence of natural enemies. Parasitoids were collected in Puerto Rico and released in India and Sri Lanka in 2009–2010, producing first year benefits to farmers of the order of $300 million (and preventing spread to Northern India). By this time, PM had spread to Thailand and the Philippines, and then was discovered in Ghana. It then rapidly spread 4000 km along the coasts of West and Central Africa. The pest’s preferred host is papaya, but it is highly polyphagous, feeding on 80 other species. Parasitoids were released in West Africa in 2013. In SE Asia, it has spread to mulberry, cassava, tomato, and eggplant.

The cassava pink mealybug was first reported in the greater Mekong region of Thailand in 2008, and quickly spread to infest 200,000 hectares by 2010 [11]. The CM-IPM programme was developed with multiple tactics: ploughing and drying soil, soaking stalk cuttings in insecticide, burning of infested plants, no transport of infested plant materials, and the release of *Anagyrus* parasitoids. In 2010–2011, six million pairs of *Anagyrus* were released in Thailand, which brought the pest completely under control by 2013 (from 200,000 to 10 hectares). However, the risk of spread to other countries in the region remains high.

Old pests also return: the brown planthopper (BPH) has been called the “ghost of green revolutions past” [135]. It was the primary threat to rice in the 1960s, was a primary driver towards the development of FFS at the end of the 1980s, yet has resurfaced as a major pest threat in the 2000s owing to resistance to continued overuse of insecticides, the heavy use of nitrogen fertilizers, and changes to climate and host ranges. BPH is often triggered by overuse of insecticides, often then reinforcing farmers’ fears of insect pests, provoking in them the wish to apply more. In China, between 6–9 M ha were infested with BPH in 2005–07, up from 2 M ha in the 1990s [135]. Farmers in China apply on average 180 kg N/ha to rice, and N-enriched plants are known to enhance size, performance and abundance of herbivorous pests.

## 9. Conclusions

Crop pests, diseases and weeds pose a substantial challenge to global food security, poverty alleviation and agricultural livelihoods. Approaches of IPM provide an array of methods by which damage can be reduced. We have demonstrated that for farmers across Asia and Africa, IPM projects have been able to deliver substantial reductions in pesticide use coupled with increased yields. Reduced reliance on synthetic pesticides delivers a range of on- and off-farm benefits, including savings, improved public health and improved natural capital on and around farms.

However, IPM is much more than just a simple resource-conserving technology. As with other forms of sustainable intensification, techniques of IPM are knowledge-intensive. Farmer Field Schools and other participatory forms of knowledge development and sharing have been invaluable in IPM extension. Thus, IPM may be viewed as a form of sustainable intensification that increases synergies between social, human and natural capital. Benefits go beyond increased crop yields and, depending on the system in use, may include improved soil health, livestock integration and income diversification. These, coupled with reduced crop losses and savings of the cost of pesticides, make IPM particularly important for tropical smallholders. Policy support is critical, but has so far been patchy. Initial gains, such as those achieved by early adopters like Indonesia, may be undone without long-term support.

## Supplementary Files

Supplementary File 1
