# Supplementary material for: Integrated Pest Management for Sustainable Intensification of Agriculture in Asia and Africa
Source: Insects. 2015 Mar 5;6(1):152–82. doi: 10.3390/insects6010152 (PMC4553536; doi:10.3390/insects6010152)
Supplement: Supplementary File 1 [file insects-06-00152-s001.pdf]

Supplementary Materials

Table S1. List of projects analysed.

Project Number	Country	Programme	Reference
	**Rice**		
21	Bangladesh	National Farmer Field School	van den Berg & Jiggins 2007
53a	Bangladesh	IPM fish-rice aquaculture INTERFISH & NOPEST	Pretty & Hine, 2003, Desilles and Barzman, 2002
53b	Bangladesh	IPM fish-rice aquaculture INTERFISH & NOPEST	Pretty & Hine, 2003, Desilles and Barzman, 2002
54	Bangladesh	DAE UNDP-FAO, DANIDA-SPPS, CAD, AID	Pretty & Hine, 2003, Community IPM
55a	Bangladesh	UBINIG ecological agriculture	Rasul & Thapa, 2003
55b	Bangladesh	UBINIG ecological agriculture	Rasul & Thapa, 2003
56	Bangladesh	Proshika ecological agriculture	SAFE2, Proshika reports
23	Cambodia	National Farmer Field School	van den Berg & Jiggins 2007
6a	China	National Farmer Field School	Mangan and Mangan 1998
6b	China	National Farmer Field School	Mangan and Mangan 1998
57	China	Rice aquaculture, Jiangshu	Pretty & Hine 2001, Li 1998
58	China	Rice mixtures, Yunnan	Zhu et al, 2000; Wolfe, 2000
59	China	Rice FFS & community IPM	Pretty & Hine, 2001, 2003
61	India	MS Swaminathan - Ecotech centres	Pretty & Hine, 2001, MSSRF
62	India	Rice FFS and community IPM	Pretty & Hine, 2001, FAO
12	Indonesia	National Farmer Field School	Kartaatmadja et al 1994
13	Indonesia	National Farmer Field School	Winarto 2004
14	Indonesia	National Farmer Field School	Feder et al 2004
18	Indonesia	National IPM	van den Berg & Jiggins 2007
24a	Indonesia	National Farmer Field School	van den Berg & Jiggins 2007
30	Indonesia	National IPM	van den Berg & Jiggins 2007
63	Indonesia	Rice FFS and community IPM	Pretty & Hine 2001, Dilts, 1998
47	Japan	Mokichi Okada Association Natural farming	Pretty & Hine, 2001: Shoji Mizuno
60	Laos	Rice FFS & community IPM	Pretty & Hine 2003, FAO
1	Nepal	National Farmer Field School	Bartlett 2005
7	Philippines	National Farmer Field School	Price 2001
11	Philippines	National Farmer Field School	Palis et al 2006
17	Philippines	National IPM	Ketelaar & Abubakr 2012
48a	Philippines	IPM rice - farmer field schools	Heong & Escalada 1998
48b	Philippines	IPM rice - farmer field schools	Heong & Escalada 1998
49	Philippines	MASIPAG rice farming	SAFE 1 & 2 - E Atega
5	Sri Lanka	FFS	Tripp et al 2005
25	Sri Lanka	FFS National IPM programme	van den Berg & Jiggins 2007
26	Sri Lanka	FFS 2003	Tripp et al 2005
50a	Sri Lanka	IPM in vegetables and rice, CARE	Jones, 1999
51	Thailand	NE Famer local wisdom networks	Pretty & Hine 2003, Ruansoongnern, 2003
15	Thailiand	National Farmer Field School	Praneetvatakul & Waibel 2006
8a	Vietnam	National Farmer Field School	Huan et al 1999
8b	Vietnam	Rice heuristic in media	Huan et al 1999
16a	Vietnam	FFS	Ketelaar & Abubakr 2012

Table 1. *Cont.*

16b	Vietnam	FFS	Ketelaar & Abubakr 2012
19	Vietnam	IPM 1995	van den Berg & Jiggins 2007
52a	Vietnam	National Inst Plant Protection	Pretty & Hine 2001, Nguyen Van Tuat
39a	Mali	FAO FFS programme	Settle and Hama Garba, 2011
42	Mali	SRI	Styger et al 2011
39b	Senegal	FAO FFS programme	Settle and Hama Garba, 2011
	**Maize**		
64a	China	East Gansu Sustainable Ag	Pretty & Hine 2001, Fan Tinglu, Drylands FI
65	Nepal	Community Welfare & Development Society	Pretty & Hine 2001
85	Ghana	IPM FFS	Montpellier Panel
2	Kenya	National FFS programme	Davis et al 2005
38a	Kenya	Push pull	Khan et al, 2012
44	Malawi, Tanz + 3	Fertlizer trees & Striga control	Ajayi et al 2011
3	Tanzania	National FFS programme	Davis et al 2005
45	Tanzania	Conservation agriculture	Kassam et al, 2009
4	Uganda	National FFS programme	Davis et al 2005
	**Wheat**		
55d	Bangladesh	UBINIG ecological agriculture	Rasul & Thapa, 2003
67	Bangladesh	Minimum tillage wheat	Pretty & Hine 2001, CIMMYT
64b	China	East Gansu Sustainable Ag	Pretty & Hine 2001, Fan Tinglu, Drylands FI
66a	China	Xiji experimental watersheds	Pretty & Hine 2001, Beijing Foresty Univ
	**Sorghum/Millet**		
68a	India	WOTR, Maharashtra	Pretty & Hine, 2001, WOTR
68b	India	WOTR, Maharashtra	Pretty & Hine, 2001, WOTR
38b	Kenya	Push pull	Khan et al, 2010, 2011
40	Mali, BF, Niger	Pearl millet IPM & FFS	Payne et al, 2011
	**Vegetables**		
22a	Bangladesh	IPM FFS	van den Berg & Jiggins 2007
22b	Bangladesh	IPM FFS	van den Berg & Jiggins 2007
22c	Bangladesh	IPM FFS	van den Berg & Jiggins 2007
37a	Bangladesh	IPM FFS	van den Berg & Jiggins 2007
37b	Bangladesh	IPM FFS	van den Berg & Jiggins 2007
73	Bangladesh	BRAC vegetable cultivation programme	Pretty & Hine 2001, Md A Saleque, Abed, 1997
33	India	Shallot onion IPM package	Dinukin et al., 2013
35a	Nepal	IPM vegetable package	IPM Innovation Lab, 2014
35b	Nepal	IPM vegetable package	IPM Innovation Lab, 2014
69	Philippines	Luzon IPM	Cuyno et al, 2001
70a	Philippines	IPM & FFS for highland vegetables, CABI	Pretty & Hine 2001, CABI
70b	Philippines	IPM & FFS for highland vegetables, CABI	Pretty & Hine 2001, CABI
50b	Sri Lanka	IPM in vegetables and rice, CARE	Jones, 1999
50c	Sri Lanka	IPM in vegetables and rice, CARE	Jones, 1999
31a	Vietnam	Vegetable IPM	ADDA 2001
31b	Vietnam	Vegetable IPM	ADDA 2001
31c	Vietnam	Vegetable IPM	ADDA 2001

Table 1. *Cont.*

52b	Vietnam	National Inst Plant Protection	Pretty & Hine 2001, Nguyen Van Tuat
72	Vietnam	Rice FFS and community IPM	Pretty & Hine 2003
86	Vietnam	Vinh Phuc Prov veg IPM	P M Sang, 2002
32a	Burkina Faso	FAO FFS programme	Settle and Hama Garba 2011
32b	Burkina Faso	FAO FFS programme	Settle and Hama Garba 2011
32c	Burkina Faso	FAO FFS programme	Settle and Hama Garba 2011
34	Mali & Senegal	IPM and FFS	Settle and Hama Garba 2011
36	Uganda	IPM and whitefly package	IPM Innovation Lab 2014
	**Potato/Sweet potato**		
55c	Bangladesh	UBINIG ecological agriculture	Rasul & Thapa, 2003
66b	China	Xiji experimental watersheds	Pretty & Hine 2001, Beijing Foresty Univ
74a	Indonesia	Sweet pot IPM & FFS (VECO-CIP-UPWARD)	Pretty & Hine 2001, Suharto et al, 2002, Johnson et al, 2001
74b	Indonesia	Sweet pot IPM & FFS (VECO-CIP-UPWARD)	Pretty & Hine 2001, Suharto et al, 2002, Johnson et al, 2001
50d	Sri Lanka	IPM in vegetables and rice, CARE	Jones, 1999
43	Uganda	Package	Roothaert 20111
	**Soybean/Bean**		
24b	Indonesia	National Farmer Field School	van den Berg & Jiggins 2007
83	Uganda	PABRA bean rot IDM	Jones et al 2011
	**Cotton/Tea**		
27a	China	FFS	van den Berg & Jiggins 2007
27b	China	FFS	van den Berg & Jiggins 2007
10	India	FFS	Mancini 2006
28	India	FFS	van den Berg & Jiggins 2007
80	India	Eco-friendly cotton, Karnataka	Pretty & Hine 2003
81	India	Maikaal cotton	Pretty & Hine 2001
29	Pakistan	FFS 2003	Khan et al 2003
82	Pakistan	Learning by doing - Zeneca	Pretty and Hine 2001, P Guest
20a	Vietnam	IPM Phutho	van den Berg & Jiggins 2007
20b	Vietnam	IPM Thai Nguyen	van den Berg & Jiggins 2007
76	Egypt	Cotton IPM - KfW	Pretty & Hine 2001
77	Egypt	Biodynamic cotton	Pretty & Hine 2001, Saber 1999
41	Kenya	FFS Lipton tea	Mitei 2011
9	Mali	FFS	Settle et al 2104
85	Mali	Organic cotton	Traore and Bickersteth, 2011
78	Senegal	Organic cotton	Pretty & Hine 2001
79	Tanzania	Organic cotton	Pretty & Hine 2001
46	Zimbabwe	FFS	Mutunda & Mpangwa
75	Zimbabwe	Zimbabwe IPPM programme	Musvoto, 2002
